# Circulating biomarkers in patients with glioblastoma

**DOI:** 10.1038/s41416-019-0603-6

**Published:** 2019-10-31

**Authors:** Juliana Müller Bark, Arutha Kulasinghe, Benjamin Chua, Bryan W. Day, Chamindie Punyadeera

**Affiliations:** 1 Saliva and Liquid Biopsy Translational Research Team, The School of Biomedical Sciences, Institute of Health and Biomedical Innovation, Queensland University of Technology, Kelvin Grove, QLD 4059 Australia; 2 Translational Research Institute, Woolloongabba, QLD 4102 Australia; 3 Faculty of Medicine, University of Queensland, 288 Herston Road, Herston, QLD 4006 Australia; 4 Cancer Care Services, Royal Brisbane and Women’s Hospital, Herston, QLD 4029 Australia; 5 School of Biomedical Sciences, Faculty of Health, Queensland University of Technology, Gardens Point, QLD 4000 Australia; 6 Cell and Molecular Biology Department, Sid Faithfull Brain Cancer Laboratory, QIMR Berghofer MRI, Brisbane, QLD 4006 Australia

**Keywords:** CNS cancer, Tumour biomarkers

## Abstract

Gliomas are the most common tumours of the central nervous system and the most aggressive form is glioblastoma (GBM). Despite advances in treatment, patient survival remains low. GBM diagnosis typically relies on imaging techniques and postoperative pathological diagnosis; however, both procedures have their inherent limitations. Imaging modalities cannot differentiate tumour progression from treatment-related changes that mimic progression, known as pseudoprogression, which might lead to misinterpretation of therapy response and delay clinical interventions. In addition to imaging limitations, tissue biopsies are invasive and most of the time cannot be performed over the course of treatment to evaluate ‘real-time’ tumour dynamics. In an attempt to address these limitations, liquid biopsies have been proposed in the field. Blood sampling is a minimally invasive procedure for a patient to endure and could provide tumoural information to guide therapy. Tumours shed tumoural content, such as circulating tumour cells, cell-free nucleic acids, proteins and extracellular vesicles, into the circulation, and these biomarkers are reported to cross the blood–brain barrier. The use of liquid biopsies is emerging in the field of GBM. In this review, we aim to summarise the current literature on circulating biomarkers, namely circulating tumour cells, circulating tumour DNA and extracellular vesicles as potential non-invasively sampled biomarkers to manage the treatment of patients with GBM.

## Background

Gliomas are the most common type of tumours originating from the central nervous system (CNS) and can be classified according to the cells that give rise to them: oligodendrocytes give rise to oligodendrogliomas, ependymal cells generate ependymomas and astrocytes produce astrocytomas.^1^ Astrocytomas can be further classified according to the WHO definitions, based on the degree of malignancy, ranging from grade I to IV; grade IV tumours are also called glioblastomas (GBM).^1^ Among astrocytomas, GBM is the most frequent and fatal form; the incidence rate in the United States is 3.20 per 100,000 population, and GBM accounts for 60–70% of malignant gliomas.^2,3^ On the basis of genome, transcriptome and proteome profiling, Phillips et al.^4^ have categorised GBM into three molecular subtypes: proneural, classic and mesenchymal.^4–8^ Each subtype shows important genetic changes, reflecting altered signalling pathways, leading to differences in treatment responses, ultimately affecting a patient’s prognosis.^4^

Current therapeutic modalities for GBM consist of a combination of surgery (which aims for maximal resection of the tumour), radiotherapy and chemotherapy. The standard chemotherapeutic drug used is temozolomide (TMZ).^9,10^ However, even with TMZ, patients have a low median survival of ~15 months.^10^ In addition, GBM patients show high rates of resistance to therapies and high rates of relapse, which result in poor overall survival.^10–14^ Some immunotherapies, such as nivolumab, which targets the immune checkpoint molecule programmed cell protein 1 (PD-1), and bevacizumab, which targets vascular endothelial growth factor, are being trialled to improve treatment in GBM patients.^15–17^ Early data have shown benefit from the use of a PD-1 inhibitor, in other tumour types, as melanoma^18,19^ and non-small-cell lung cancer^20^ in patients with a high tumour mutational burden. However, GBM is thought to have a low mutational burden and to be immunologically cold.^21^ Moreover, other studies have reported that immunotherapies can alter the tumour microenvironment in GBM, which may influence patients’ response to treatment and culminate in benefits from combined therapies.^15,22^

The diagnosis of GBM is currently based on imaging techniques and tissue biopsies.^9^ However, imaging techniques cannot reliably differentiate lesions caused by actual tumour progression from pseudoprogression—treatment-related lesions that mimic tumour progression and might resolve spontaneously over time. Similarly, tissue biopsies entail a highly invasive procedure, yet might only capture a static snapshot of an ever-changing tumour.^23^ By contrast, liquid biopsies that enable the detection of circulating biomarkers confer the benefit of being non-invasive, thereby facilitating serial sampling and the ability to monitor potential dynamic changes in the tumour over the course of therapy.^23–25^

Tumours in general, including GBM, shed tumoural content into the blood^26^ and cerebrospinal fluid (CSF).^27^ The detection of these biomarkers, such as proteins, cell-free nucleic acids (cfNAs), extracellular vesicles (EVs) and circulating tumour cells (CTCs), in a liquid biopsy can be used to complement standard risk-stratification methods, monitoring of treatment response and disease progression in GBM patients. This review aims to summarise the current literature on circulating biomarkers that are found in the blood of GBM patients, with a focus on CTCs, circulating tumour DNA (ctDNA) and EVs.

## Current approaches to the management of GBM

### Diagnosis

The initial diagnosis of GBM is achieved by neuroimaging, followed by resection or biopsy of tumour tissue to definitively diagnose, grade and characterise the tumour. Currently, tissue biopsies are the gold-standard technique for GBM diagnosis. However, resection or biopsy from a brain tumour can present risks to the patients, such as possible brain swelling within and around the tumour mass, or might even affect neurological functions.^28^ Moreover, some tumours might be inaccessible owing to their location.^29^ Furthermore, tissue biopsies can sometimes fail to predict the heterogeneity of the whole tumour mass and might not be a true representation of the tumour activity in real time.^23^

Further confirmatory and descriptive tests are performed on tumour samples by using immunohistochemistry and molecular analyses,^16,30–32^ including the combined loss of chromosome arms 1p and 19q, the mutation and/or expression of p53, the presence of isocitrate dehydrogenase 1 (IDH1) mutation (within exon 4 to codon 132, the most common being c.395 G > A (R132H) substitutions^33^) and epigenetic alterations, such as O^6^-methylguanine-DNA methyltransferase (MGMT) hypermethylation.^9,32^

### Treatment

As alluded to above, current therapeutic modalities for GBM entail a combination of surgery followed by radiotherapy and/or chemotherapy. In surgery, it is challenging to safely remove all tumour cells due to the high invasive capacity of GBM cells into normal tissue; as a result, GBM tumours recur in the majority of the cases.^34^ In patients with recurrent GBM, the median overall survival is 6.2 months.^34^

### Prognosis and pseudoprogression

**Fig. 1:**
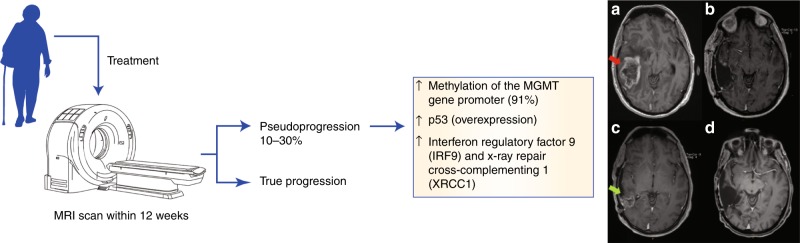
Pseudoprogression. After treatment, a brain MRI scan is performed in GBM patients. When the MRI is performed within 12 weeks of treatment, 10–30% of patients may present enhanced lesions that may improve with time, which are known as pseudoprogression. A correlation between the methylation of the MGMT gene promoter, and overexpression of p53, IRF9 and XRCC1, and the occurrence of pseudoprogression has been observed.^35–37^ Example of pseudoprogression in a male patient diagnosed with glioblastoma at 51 years of age. Initial gadolinium-enhanced T1-weighted MRI prior to any treatment (**a**) demonstrated a heterogeneously enhancing right temporal mass (red arrow), which was resected; histopathology was consistent with glioblastoma. Immediate postoperative imaging (**b**) demonstrated near-complete resection of tumour, but MRI after adjuvant radiation and chemotherapy (**c**) was concerned with progression due to interval development of new irregular enhancement adjacent to the cavity (green arrow). On this basis, he proceeded to further debulking surgery, the histopathology consistent with necrosis only. Five years after diagnosis, his MRI (**d**) remained free of evidence of recurrence, consistent with the diagnosis of pseudoprogression. Figure produced using Servier Medical Art

To obtain prognostic information, a brain MRI scan is performed after treatment. Contrast-enhancing lesions that appear on the images can be caused by tumour progression, but might also be due to post-radiotherapy changes, referred to as pseudoprogression, which might resolve spontaneously^35^ (Fig. 1). Pseudoprogression occurs in 10–30% of GBM patients who have had their first MRI scan, usually within the first 12 weeks of treatment.^35^ The ability to differentiate between pseudoprogression and true progression is important, as it would help clinicians to avoid performing unnecessary operations and prescribing ineffective therapies.^25,28,30,35,36^ However, currently, there are no biomarkers or clinical features to distinguish glioma true progression from pseudoprogression. Brandes et al.^37^ showed, by using tissue biopsy samples from 103 patients with GBM, that patients with methylation of the MGMT gene promoter had higher rates of pseudoprogression (91%) than patients with unmethylated MGMT (41%), as shown by MRI. In their study, pseudoprogression was found in 31% of patients.^37^ In addition, Kang et al.^38^ discovered that p53 overexpression in tumour tissue sections correlated with pseudoprogression in 35 tumour samples from glioma patients. p53 was considered to be overexpressed when >10% of the tumour cells stained positive for p53, and of the 13 patients whose samples showed p53 overexpression, seven had pseudoprogression, three presented with non-progression and three showed early progression. Pseudoprogression rates were higher in glioma patients who presented with p53 overexpression than in patients without p53 overexpression. In a total of 22 patients whose samples did not show p53 overexpression, 14 showed non-progression, one presented with pseudoprogression and seven presented with early progression.^38^ Following their analysis of tumour tissue from 17 glioma patients to identify a potential biomarker for pseudoprogression, Qian et al.^39^ suggested that higher expressions of interferon regulatory factor 9 (IRF9) and X-ray repair cross-complementing 1 (XRCC1) were associated with pseudoprogression. However, despite these emerging data, more studies in this field are warranted to identify a biomarker that can be implemented into a clinical setting to better differentiate true progression from pseudoprogression.

In order to improve outcomes for patients with GBM new and merging strategies are used. These include non-invasive methods of sampling to help in the diagnosis and monitoring of GBM. Some of the efforts are in the fields of advanced magnetic resonance imaging and spectroscopy (MRI/MRS), plasma immunoprofiling and liquid biopsies. Currently, conventional MRI is a gold-standard technique for workup and treatment response, providing detailed structural information enabling guided surgery and mapping of tumour tissues. Nevertheless, in the initial workup, this technique cannot distinguish between different high-grade gliomas, such as glioblastoma from oligodendroglioma,^40^ and there may also be difficulties in distinguishing infective causes, lymphoma and metastases from primary brain tumours.^41^ After treatment of GBM, there remain significant imaging challenges in response assessment, such as pseudoprogression and pseudoresponse.^40^ Advanced MR techniques are being investigated to provide more detailed information on tumour properties, but many of these remain investigational.^40,42^ MR may also not predict molecular characteristics of primary brain tumours, such as MGMT methylation and IDH mutation status. Han et al.^43^ correlated the MGMT promoter methylation with tumour location and necrosis by using advanced MRI. However, Moon et al.^44^ did not find significant association between MGMT methylation status and tumour location. Furthermore, small cohort sizes were used in these studies. Therefore, there is a need for more research to be conducted to better understand the role of advanced MR imaging.^40^

## Liquid biopsies

Tumours shed their tumoural content into circulation and can be sampled in a number of body fluids.^45^ Examples of these are CTCs, cfNAs, ctDNA and EVs (comprising both microvesicles [MVs] and exosomes). The majority of these biomarkers have a short half-life, though—up to 3 h—and are rapidly degraded when present freely in the plasma.^25,46^ However, some of them are packaged in EVs, such as MVs and exosomes, which offers protection from degradation by circulating proteases and nucleases.^25^

The sampling and analysis of these molecules in non-solid biological fluids is defined as a liquid biopsy,^47^ fluid biopsy or fluid-phase biopsy.^48^ Although liquid biopsies are often carried out by using a blood draw, other biofluids, such as saliva and urine, can be also used.^49^ Cerebrospinal fluid (CSF) has also been used to investigate tumour-specific biomarkers in brain tumours,^27,50^ as it circulates along with the brain and spinal cord, and therefore has close contact with the CNS, but CSF collection requires an invasive lumbar puncture procedure. Liquid biopsy presents a minimally invasive way to capture tumour activities in real time to diagnose and predict disease progression.^49,51^

The use of liquid biopsies has been studied in different tumour types, and this approach has been used in the prognosis of breast cancer,^52^ head and neck^53^ and lung cancer.^54^ For lung cancer, for example, when tumour tissue is limited, blood plasma can be used to detect the presence of mutations in the epidermal growth factor receptor.^55,56^ The first FDA-approved broad companion pan-cancer diagnostic test utilises the presence of ctDNA within a liquid biopsy sample to test for the presence of a number of solid tumours, including non-small-cell lung cancer, colorectal cancer, breast cancer, ovarian cancer and melanoma.^57,58^

For liquid biopsies to be successfully used in GBM, it is assumed that tumour-specific material would cross the blood–brain–barrier (BBB). The BBB regulates the access and exchange of nutrients, vitamins and other molecules into the brain.^59^ The integrity of the tight junctions of the BBB, which is determined by proteins such as claudin-3, claudin-5, claudin-12 and other transmembrane proteins with scaffolding functions, defines the quality of a healthy BBB,^60^ and accordingly, a deficiency or mutation in claudin-1 or agrin has been related to BBB dysfunction in GBM.^59^ GBM induces a proangiogenic and inflamed microenvironment, which decreases tight junctions, helping to establish a more permeable BBB, supporting the access of pro-inflammatory immune cells such as tumour-associated macrophages.^61^ In addition, hypoxia, a typical feature of solid tumours such as GBM, is also correlated with the disruption of the BBB.^62^

Zhao et al.^62^ reported that exosomes derived from the hypoxic GBM cell line U87 promoted the proliferation of brain microvascular endothelial cells (BMVECs), inducing BBB permeability in an in vitro model. Changes in BBB permeability are seen within different stages of gliomas, as the progression of the disease aggravates its disruption and permeability increases.^63^ This disruption can be seen on MRI by using a contrast medium such as gadolinium, which does not normally cross the intact BBB.^63,64^ However, some regions of GBM can have an intact BBB.^65^ Despite the association between BBB dysfunctions and GBM disease progression, EVs derived from glioma cells were shown to cross the intact BBB and were detected in the blood of GBM patients.^66^ This highlights the importance of EVs in liquid biopsies since EVs can be detected in cases in which the BBB is not compromised.

**Fig. 2:**
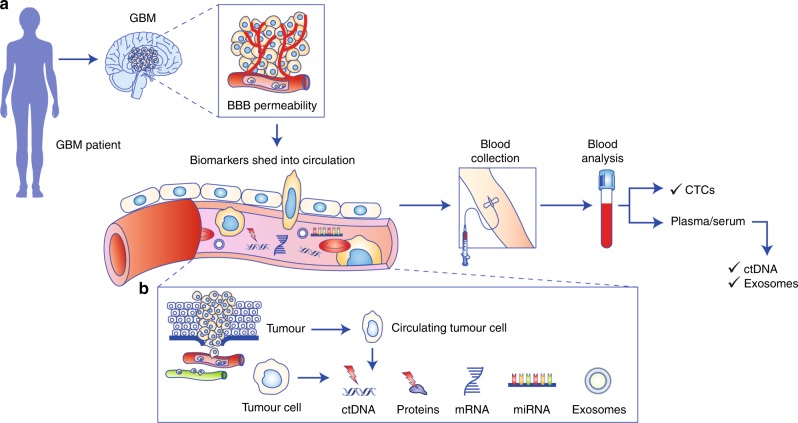
A schematic representation of biomolecular transportation from a tumour through the BBB into the circulation. (**a)** In patients with GBM, a leaky BBB allows circulating biomarkers—for example, circulating tumour cells (CTCs), circulating tumour DNA (ctDNA) and microvesicles—to enter the circulatory system, from where they can be collected, via blood draw, and further analysed. (**b)** A breakdown of the tumoural components found in the circulatory system. Several classes of biomarkers can be accessed and measured in liquid biopsies, including CTCs, which can be shed from a primary tumour; extracellular vesicles, which can be released by tumour cells (and can carry nucleic acids and proteins inside); ctDNA, which can also be released by tumour cells. These molecules carry tumoural information (e.g., mutational status, tumoural cargo), which can be sampled non-invasively. Figure produced using Servier Medical Art

Liquid biopsy could therefore be a helpful tool to complement current strategies for predicting GBM prognosis, by allowing a more dynamic view of tumour characteristics, and response to chemotherapy, by providing a platform (through multiple sampling) to monitor treatment responses.^49,51,67^ A schematic illustration of circulating biomarkers that could be investigated in GBM patients’ blood is shown in Fig. 2; these biomarkers are discussed in more detail below.

## Circulating tumour cells

### CTCs and metastasis

Glioma metastasis outside of the CNS is a very rare event, with ~0.4–0.5%^26,68^ of gliomas metastasising to sites such as the lungs and pleura, regional lymph nodes, bones and liver.^69^ The low rates of distant metastasis might be due to low survival rates of GBM patients, or a possible suppression of tumour cell growth outside the CNS by the immune system or the BBB, the presence of which makes it more difficult for the cells to intravasate into the circulation. Despite these low rates, some cases of extracranial metastases have been reported when patients have signed up to donate organs, providing evidence that distant metastasis can occur with glioma patients.^26,68,70^

In several solid tumour types, metastasis is normally related to the presence of CTCs—cells that are shed by either primary or secondary tumours into the circulatory system.^71,72^ CTCs are metastatic precursor cells that undergo epithelial–mesenchymal transition (EMT), a cellular process that is characterised by a more mesenchymal phenotype and increased migratory potential. CTCs can then extravasate from the circulatory system and colonise other organs. CTCs can be released as single cells, or in homotypic or heterotypic clusters, which have been reported to have a higher metastatic propensity compared with single CTCs.^73–76^ Szczerba et al.^64^ reported that CTC–neutrophil clusters injected into tumour-free mice induced faster tumour formation than single CTCs, as well as increased metastatic potential and shorter overall survival.^76^ Gkountela et al.^74^ observed in breast cancer that the methylation status of CTC clusters is linked with the prognosis of the disease. Transcription factor binding sites in genes related to cell stemness and proliferation were differentially methylated in CTC clusters (hypomethylated) in comparison with single CTCs (hypermethylated), and this alteration is reflected by an increased stemness phenotype and metastatic ability, culminating in a poor prognosis.^74^ CTCs have been detected and characterised in different tumour types, and their presence has been found to correlate with poor overall survival.^77,78^ However, CTC research in GBM is limited: the first study on CTCs was published in 2014,^26,68,79^ and the first paper on CTC clusters in GBM was published in 2018.^80^

### Isolation and characterisation of CTCs

CTCs can be isolated by using different approaches. One such approach is to use label-free selection with specific protein markers to select or to deplete those cells that express a particular marker.^47,81^ Other approaches to CTC isolation rely on differences in their physical property and use techniques, such as filtration, chip technology, density gradient centrifugation, electric field, sound waves^82^ and microfluidic technology.^54,83–85^

Characterisation of CTCs can be performed by using immunocytochemistry, molecular technologies and/or functional assays.^47^ Currently, the only FDA-approved platform for the isolation of CTCs is the CellSearch® system (Menarini Silicon Biosystems, Italy), which relies on the positive selection of tumour cells overexpressing an epithelial cell adhesion marker, EpCAM.^45,86^ Cells from GBM tumours tend to adopt a more mesenchymal phenotype compared with the epithelial nature of cells needed for detection by using the CellSearch.^68^ Therefore, alternative approaches for the isolation of CTCs need to be explored in GBM patients.

### CTCs in glioblastoma

Muller et al.^26^ detected CTCs in the peripheral blood of 29 out of 141 GBM patients (20.6%). Following their isolation by using density-gradient centrifugation, CTCs were stained for glial fibrillary acidic protein (GFAP) as a GBM marker, as well as verifying amplification of the EGFR gene and demonstrating gains and losses in genomic regions of chromosomes 7 and 10. The authors also used single-cell genomic analysis to identify common mutations found in CTCs and in tumour tissue, to prove that the CTCs were most likely derived from GBM.^26^ Similarly, MacArthur et al.^79^ isolated CTCs from glioma patients by density-gradient centrifugation by using the OncoQuick® system (Greiner Bio-One, Frickenhausen, Germany). As telomerase activity is elevated in tumour cells but not in normal cells, a telomerase-based test was used to detect CTCs, as well as testing for nestin expression as a glioma cell marker. CTCs were detected in eight out of 11 (72%) patients prior to radiotherapy, whereas the detection rate for post-radiotherapy patients was one out of 8 (8%).^79^ Sullivan et al.^68^ showed evidence of CTCs in 39% of peripheral blood samples from patients with GBM. In this study, CTC–iCHIP technology was used to enrich for CTCs by depleting haematopoietic cells from blood specimens. Interestingly, higher counts of CTCs were identified in patients with a progressive disease rather than in patients with stable disease. Positive CTCs were then characterised by using a ‘cocktail’ of antibodies against SOX2, tubulin β-3, EGFR, A2B5 and c-MET based on GBM biomarkers identified in the literature, before the expression of 25 genes, representing all the molecular subtypes of GBM (proneural, neural, classical and mesenchymal), was assessed; the results of the analysis concluded that CTCs from GBM show more of a mesenchymal phenotype.^68^ This phenotype is associated with a higher invasion capacity, allowing cells to intravasate into the circulation, which may explain the rare cases of extracranial metastases in GBM.

Gao et al.^87^ used a matrix for separation followed by a negative depletion of white blood cells by immunomagnetic beads. A polyploidy chromosome-8-positive detection was used as a positive criterion for CTCs, along with GFAP-positive or -negative cells and CD45-negative cell status to confirm glioma origin. CTCs were detected in peripheral blood from 24 out of 31 (77%) patients with seven different subtypes of glioma, including astrocytomas, oligodendrogliomas and oligoastrocytomas. In GBM patients specifically, CTCs were detected in nine out  of 11 patients (82%). No correlation was found between the number of CTC and the different grade of glioma, but interestingly, when the authors investigated patients with new enhancing mass lesions (five patients), the results correlated with CTC counts. Of the five patients in total, three had GBM. Two of these GBM patients had CTC counts of two and three, respectively, and showed recurrence of the disease; the CTC count of the other patient that showed possible recurrence by imaging was zero. As the patient presented with no clinical symptoms, no treatment was given. The patient was asked to return after 1 month, whereupon the new imaging results showed a decrease in enhancing lesion, which is indicative of pseudoprogression and not true progression.^87^

In 2018, Krol et al.^80^ observed the first evidence of CTC clusters in GBM and could detect CTCs in seven out of 13 patients with progressive GBM (53.8%). Blood collections were made at seven different time points during disease progression in an open-label Phase 1/2a study testing the compound BAL101553 (a microtubule inhibitor), and CTCs were isolated by Parsortix microfluidic technology.^88^ The authors identified positive CTCs as cells that met at least one of the following criteria: they were at least 9 µm in size, and were negative for CD45 staining; they were positive for EGFR, Ki67 or the microtubule-associated protein EB1, as well as being CD45 negative. Liu et al.^89^, also in 2018, isolated CTCs from GBM patients as previously described^79^ and characterised their stemness by immunohistochemistry by using Olig2 and CD133.^89^ A mouse model was used to show the capacity of CTCs to reseed the primary tumour site when injected intravenously. Also, by using cell viability and apoptosis assays, the authors analysed the resistance of CTCs to radiotherapy and chemotherapy with TMZ and concluded that CTCs are more resistant to treatments and to stress induced in the circulation than other tumour cells. Malara et al.^90^ reported a case of a 67-year-old patient presenting with an intracranial lesion that was subsequently confirmed to be GBM. The patient’s blood was collected before surgery and 2 months post-operation, and CTCs were captured by using density-gradient centrifugation. The first blood sample showed 4.5 CTCs/ml of blood, but this figure increased to 7 CTCs/ml in the second sample. After 9 months, the patient presented with tumour recurrence, and 5 months later had succumbed to the disease.^90^ Along with imaging results, CTC analysis proved to be relevant in monitoring the patient’s intracranial lesion.

The number of studies showing the detection of CTCs in GBM patients is still limited, and the use of different approaches to isolate and characterise these cells makes it difficult to compare their results. Also, the number of patient samples remains a limitation. However, these studies demonstrate an increased application of CTCs in GBM studies with the potential for investigation in clinical trials, but clearly, larger trials are warranted.

## Circulating tumour nucleic acids

Cells may release DNA and RNA (including mRNA and non-coding RNA) content into the circulation. Cell-free DNA (cfDNA) comprises small fragments of DNA (180–200 base pairs) released by cells under physiological and pathological conditions. It is suggested that the main source of cfDNA derives from apoptotic cells.^49^ When released by normal cells, these fragments are generally cleared by phagocytosis, and consequently, cfDNA levels are typically low in healthy individuals.^23^ In cancer patients, a proportion of cfDNA will comprise ctDNA. The amount of ctDNA varies and is thought to reflect the burden of disease, with greater amounts of ctDNA present in more advanced/late-stage patients.^46^ ctDNA might carry tumour-specific mutations, reflecting the mutational landscape of the primary tumour, and therefore represents an important means by which to sample tumour tissue non-invasively.^75^ However, there are challenges associated with ctDNA analysis, such as the sensitive detection technologies needed to distinguish mutant from wild-type alleles and the development of thresholds for mutations (e.g., variant allele frequency (VAF)). Furthermore, ctDNA fragments present a short half-life of <1.5 h^23^ and require to be processed rapidly. Tumour cells can also shed different classes of RNA into the circulation, such as protein-coding mRNA, and non-coding microRNAs (miRNA) that are small and contain around 21–24 nucleotides, and long non-coding RNAs (lncRNA) that consist of 200 nucleotides or greater. These noncoding RNAs play a significant role in gene regulation and can be found as circulating cell-free nucleic acids or inside of EVs, which provide more stability. Circulating RNAs have been found in blood and CSF of glioma patients, and may act as biomarkers for prognosis, diagnosis and treatment monitoring.^24,91,92^

### ctDNA in glioblastoma

Schwaederle et al.^93^ conducted a study in which ctDNA in plasma samples collected from 171 patients with different tumour types was analysed on a targeted panel (54-gene panel) by using next-generation sequencing (NGS). Of these 171 patients, 33 had GBM diagnosis (representing 19% of samples). Unaltered ctDNA was detected in 73% of the patients with GBM; 24% had one alteration, and 3% had two or more alterations.^93^ In a separate study, Bettegowda et al.^46^ detected ctDNA in <10% of 27 patients with glioma by using PCR (ten out of the 27 were diagnosed with GBM). Glioma was the tumour type with the lowest frequency of cases with detectable ctDNA.^46^ These studies demonstrate a low yield of ctDNA in glioma patients’ blood, mostly justified by the presence of the BBB. By contrast, by using a NGS panel, Piccioni et al.^94^ analysed 419 patients with primary brain tumours, including 222 patients with GBM, and detected ctDNA mutations in blood samples collected from 50% of all brain-tumour patients—55% among the GBM patients. The authors concluded that for patients with detectable ctDNA levels, the results of plasma ctDNA analysis could provide a viable option to pursue treatment alternatives.^94^ If ctDNA is detected, specific mutations can be tracked during treatment, which might reveal an increase in tumour-specific DNA or a change in DNA methylation status. The DNA methylation status can indicate acquired resistance to a certain treatment, and therefore tracking those alterations could guide the modification of treatment.^95^ When Wang et al.^96^ analysed the serum and CSF of patients with different grades of glioma, they detected the presence of methylation in the MGMT promoter, by using methylation-specific PCR, in 38 patients with GBM out of 89 glioma patients (42.6%). This analysis showed a higher sensitivity when using CSF instead of blood samples (serum), with MGMT promoter methylation detected in 19 out of 89 patients (21.3%) in serum samples, and in 26 out of 78 patients (33.3%) by using CSF.^96^ This higher sensitivity found in CSF suggests that the use of CSF may present an advantage in comparison with serum and might be due to the BBB presence that prevents ctDNA clearing.

In a pilot study conducted by Salkeni et al.,^97^ plasma samples from three out of 13 patients (23%) newly diagnosed with GBM contained the EGFRvIII deletion variant. This variant, which is frequently detected in GBM patients with EGFR amplification, contains a deletion in exons 2–7, which generates a constitutively active form of EGFR to confer activation of EGFR downstream targets. The authors suggested that the levels of EGFRvIII DNA in patients’ blood might correlate with the tumour resection status, as the levels of EGFRvIII DNA were higher in a patient who showed incomplete tumour removal.^97^ Faria et al.^98^ observed that the cfDNA levels were significantly increased (by ~30-fold) in patients with GBM or brain metastases who were receiving a treatment of intranasal administration of perillyl alcohol compared with the healthy control group. After treatment, patients who survived more than 6 months had 2.7-fold lower cfDNA levels than patients who survived <6 months.^98^ This result indicates that the relevance of cfDNA levels might present to prognostic assessment in GBM. By using a patient-derived orthotopic xenograft model, Mair et al.^99^ showed that tumour size and cell proliferation influence the release of ctDNA in mice prior to treatment, while BBB integrity does not. However, they also found that cell death post therapy is an additional factor that can augment ctDNA release. These results suggest that BBB may not play the main role in ctDNA release as it has been suggested in previous studies. More studies need to be conducted in order to define the exact contribution of BBB to ctDNA release. In addition, the authors reported that whereas ctDNA was detected in 24% of mice (15/64), plasma tumour mitochondrial DNA (tmtDNA) was detected in 82% of the animals (52/64). tmtDNA was also detected in CSF and urine, while ctDNA was not detectable in urine.^99^ This study demonstrates the potential use of tmtDNA analysis in GBM, and moreover, helps to elucidate different factors that influence ctDNA concentration in the circulation. In addition, this study highlights some advantages of the use of tmtDNA in comparison with ctDNA, such as high sensitivity of detection, high copy number present in glioma and low cost.

Zhao et al.^100^ studied the response of 66 patients with GBM recurrent to the PD-1 immune checkpoint inhibitors nivolumab or pembrolizumab. The authors analysed DNA and RNA from plasma, in the form of cfNAs, tumour tissue and clinical data. Genomic and transcriptomic analysis correlated with the patients’ response to immunotherapy: those who did not respond showed mutations in *PTEN* that are associated with immunosuppressive expression signatures, whereas responders carried mutations in components of the mitogen-activated protein kinase (MAPK) pathway.^100^ These results demonstrate that the response to immunotherapy by using PD-1 inhibitor varies according to specific molecular alterations, and the therapy may benefit a subgroup of GBM patients, suggesting a molecular and personalised selection of patients for immunotherapies.

### Circulating RNA in glioblastoma

miR-21 is an important miRNA studied in cancer, and its upregulation has been reported in the plasma^101^ and tissue^102^ of GBM patients, and associates with lower overall survival and tumour grading.^103^ Wang et al.^104^ analysed the plasma of ten GBM patients before and after therapy, and described two miRNAs, miR-128 and miR-342-3p, which are downregulated in patients when compared with healthy controls. miR-128 and miR-342-3p levels correlated with glioma grades and increased after surgery and chemoradiation, suggesting their use as biomarkers to assess tumour grading and to monitor treatment response.^104^ Zhi et al.^105^ analysed the serum of patients and established that the upregulation of miR-20a-5p, miR-106a-5p and miR-181b-5p correlated with tumour grading, and miR-19a-3p, miR-106a-5p and miR-181b-5p were linked with poor prognosis. In addition, Zhao et al.^106^ isolated miRNA from the serum of patients and described that miR-222-3p, miR-182, miR-20a-5p, miR-106a-5p and miR-145-5p correlated with poor patient outcome. Along with miRNA class, another noncoding RNA class, circulating lncRNAs, is emerging as potential cancer biomarkers.^107^ Tan et al.^108^ studied the prognostic value of a long non-coding RNA, HOX transcript antisense intergenic RNA (HOTAIR) in patients’ serum. HOTAIR is known to be overexpressed in GBM and to induce cell proliferation. Higher HOTAIR levels were detected in patients’ total serum and in exosomes when compared with healthy controls.^108^ Shen et al.^109^ also reported that high levels of HOTAIR and low levels of GAS5 in serum associated with a reduced probability of 2-year survival, suggesting its potential as prognostic biomarkers.

## Extracellular vesicles

EVs are membrane-bound vesicles that are released by cells under physiological and pathological conditions. EVs can carry cargo, such as mRNA, miRNA, DNA and cellular proteins, and they can be detected by using cell-surface markers.^28,110,111^ Previously, EVs were considered as artefacts or fragments of dead cells,^112,113^ but they are now known to play a major role in cell–cell communication,^114^ as their released cargo—such as mRNA, miRNA and angiogenic proteins—can be taken up by other cells, even from distant sites,^115^ thereby enabling genetic information, as well as proteins to be delivered to, and influencing the phenotype of recipient cells, such as endothelial cells. One of the advantages of these circulating biomarkers in liquid biopsy studies would be the protection of biomolecules within the EVs.

**Fig. 3:**
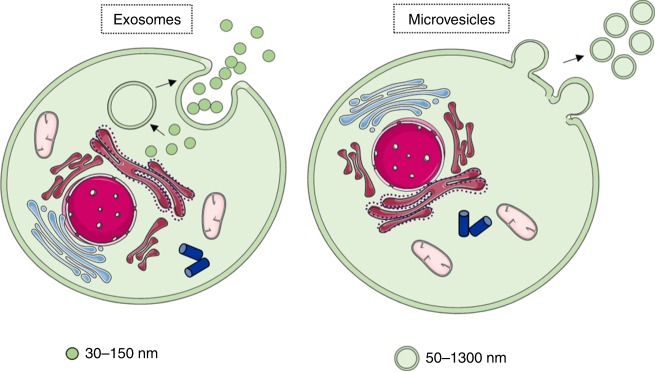
A schematic representation of the two main classes of EVs. Exosomes and microvesicles differ mainly in size and origin. The diameter of exosomes is smaller (30–150 nm), and are derived from the endosomal membrane. The diameter of microvesicles ranges from (50 to 1300 nm), and are released from cell membrane budding. Figure produced using Servier Medical Art

There are two broad types of EVs, exosomes and MVs, which differ mainly in their size and origin. Exosomes are smaller (30–150-nm diameter) and are derived from the endosomal membrane, while MVs range from 50 to ~1300 nm and are released directly from budding of the cell membrane^115^ (Fig. 3). Exosomes can be detected by transmission electron microscopy (TEM), nanoparticle-tracking analysis (NTA) and the presence of a number of membrane-associated proteins, such as CD63, CD81, CD9, CD37, CD53, CD82, ICAM-1 and integrins, all of which can be identified by flow cytometry or Western blot.^115^ Currently, there are no standard protocols in consensus to specifically isolate EV subtypes and separate exosomes and MVs. Therefore, the International Society for Extracellular Vesicles (ISEV) recommends to consider the physical/biochemical characteristics of EVs in order to name them, for example, ‘small EVs' or ‘medium/large EVs' or CD63+/CD81+ EVs.^116^ In this review, we separated the EV classes, MVs and exosomes, based on the terminology used in the original research papers.

### Microvesicles in glioblastoma

Koch et al.^117^ investigated whether blood-derived MVs could aid in differentiating GBM recurrence from tumour pseudoprogression on the basis of a difference in MV number. In blood collected from seven healthy controls or 11 patients with GBM at different treatment times, the quantity of MVs from patients with stable disease or pseudoprogression was significantly lower than in patients who underwent true tumour progression.^117^ Evans et al.^118^ also correlated an increase in MV number with poor overall survival and with earlier disease recurrence. Skog et al.,^119^ having isolated MVs from tumour samples and serum of 25 GBM patients by centrifugation, identified the EGFRvIII deletion variant in MVs from seven out of the 25 patients, whereas no EGFRvIII was detected in the control healthy group. The authors also concluded that GBM cells shed MVs, and that their content—that included angiogenic proteins in addition to EGFRvIII—can enhance the angiogenic phenotype of normal brain endothelial cells and proliferation in other glioma cells.^119^

### Exosomes in glioblastoma

Osti et al.^120^ demonstrated that the concentration of EVs was increased in GBM patients in comparison with healthy controls and patients with other CNS diseases. When comparing EV concentrations over different time points, an increase in EV concentrations correlated with tumour recurrence, suggesting that exosomes could help to predict GBM recurrence.^120^ Similarly, Andre-Gregoire et al.^121^ observed a higher concentration of EVs in patients with GBM, as well as showing that EVs from patient-derived glioblastoma stem cells, which are thought to be involved in tumour initiation, expansion, resistance to treatments and relapse, had increased cargo relating to cell adhesion after TMZ treatment, indicating that TMZ had the potential to promote the increased release of factors favouring tumour progression.^121^ Manda et al.^124^ investigated the expression of EGFR and EGFRvIII in serum exosomes and tumour tissue in 96 patients with high-grade glioma. They detected EGFRvIII in 39.5% of tumour tissue samples and in 44.7% of their paired serum exosome samples, whereas 28.1% of tumour biopsy samples had EGFR and EGFRvIII co-expression. Although the co-expression of EGFR and EGFRvIII is rare in GBM cells,^122^ this co-expression is suggested to cooperate with tumour growth and induce macrophage infiltration.^123^ Also, the presence of EGFRvIII in exosomes correlated with a lower overall survivor pattern—21.1 months—compared with 28.6 months for patients with no EGFRvIII expression in exosomes.^124^

Chandran et al.^125^ reported that syndecan-1 found in plasma EVs can be used to distinguish low-grade glioma from high-grade GBM with a sensitivity of 71% and a specificity of 80%, and provided strong support for plasma–EV-derived syndecan-1 being derived from GBM tumours. Yang et al.^126^ isolated exosomes from tumours generated in mice by using four GBM patient-derived samples collected during surgery, and reported an increase in the expression of the genes encoding dynamin-3, p65 and CD117, alongside a decrease in the expression of the genes encoding PTEN and p53, in the tumour tissue and blood of mice. In another study of 60 glioma patients, including 27 diagnosed with GBM, miRNA was detected in exosomes isolated from the serum by centrifugation and quantitated by using real-time PCR. The authors found that in comparison with low-grade gliomas, miR-301a levels were higher in high-grade gliomas. They also observed that the serum exosomal miR-301a levels were lower after surgical resection of the tumour, but were increased during GBM recurrence, indicating that serum exosomal miR-301a could be a potential biomarker for diagnosis/prognosis for GBM patients.^127,128^ Ebrahimkhani et al.^129^ used a panel of seven exosomal miRNAs—miR-182-5p, miR-328-3p, miR-339-5p, miR-340-5p, miR-485-3p, miR-486-5p and miR-543—to differentiate GBM patients from healthy controls with an accuracy rate of 91.7%. In addition, Santangelo et al.^91^ analysed a miRNA signature in exosomes from the serum of glioma patients in an attempt to differentiate tumour grading and gliomas from brain metastases. The authors demonstrated the upregulation of three miRNAs, miR-21, miR-222 and miR-124-3p in glioma. miR-21 is known to play a role in GBM pathogenesis.^130^ In their study, miR-21 differentiated healthy controls from glioma patients, but could not distinguish high-grade gliomas from other tumours’ brain metastases. Nevertheless, when in combination, the panel differentiated high-grade gliomas from other tumours’ brain metastases, suggesting that it can represent an alternative for inconclusive biopsy results or in cases in which the tumour is located in critical brain areas.^91^ Manterola et al.^131^ also analysed exosomal small non-coding RNA signature from the serum of 75 patients with GBM. The authors concluded that miR-320 and miR-574-3p, as well as a small no-ncoding RNA, RNU6-1, are upregulated and are able to discriminate GBM patients from healthy controls.^131^ Most of the EV studies in GBM present a limitation of the small size of cohorts. Therefore, there is still the need for validation of these findings in larger cohorts.

## Conclusions and future directions

There remains a need for non-invasive sampling to capture brain-tumour activity in real time to better inform prognosis of the disease and to monitor treatment responses. Current diagnosis of GBM relies on imaging and tumour tissue data; however, there are some challenges and limitations. Conventional MRI can guide surgery; however, it cannot distinguish between high-grade gliomas and may provide imaging findings that are challenging to interpret.^132^ Tumour tissue biopsies are invasive and cannot be repeated easily. Liquid biopsies present advantages when compared with the current approaches, and these include the ability to repeat sampling over the course of treatment in a non-invasive manner, and the fact that the BBB may be more permeable in the presence of a high-grade tumour, allowing molecular transportation.^63^ A liquid biopsy may be able to reveal tumour information prior to clinical progression.^133,134^ However, the tumour morphological features and the microenvironment are more readily available in the tissue biopsy. Therefore, a liquid biopsy aims to provide additional and complementary data to improve upon the diagnosis and follow-up of GBM patients.

CTCs, ctDNA and MVs have been demonstrated to be able to be sampled from different biofluids for a number of tumour types, and studies have demonstrated that these biomarkers can be found in GBM patients, and that their mutational profiles represent those of the GBM in origin. There is a pressing need to improve the technologies involved in regularly and reliably isolating and characterising these biomarkers, and larger studies in GBM investigating these biomarkers are warranted, with clinical correlatives measured over time to determine the effects on clinical outcome. Unlike for many other tumour types, the use of CTCs in GBM as a diagnostic screening tool is not ideal, because by the time a patient with GBM experiences clinical symptoms and receives a positive diagnosis from a treating physician, their disease is already at an advanced stage. However, the liquid biopsy approach shows great potential in managing GBM patients.

Currently, no clinically validated circulating biomarkers for managing GBM patients exist. One reason for the relative lack of circulating biomarkers in this field is because of the BBB, restricting the transportation of molecules from blood to the brain and vice versa. Along with biological difficulties, there are technical limitations for the establishment of a role for CTCs in GBM. Only a few studies have been carried out by using brain-tumour-derived CTCs, and they show that the detection rates vary from 20 to 77% in GBM patients, depending on the CTC isolation techniques used. However, other studies do warrant further investigation of CTCs in GBM. For example, the first report detecting CTC clusters in GBM, published in 2018, also indicates the capacity of GBM clusters to cross the BBB.^80^ This is an important clinical finding that requires large studies to test the reproducibility of these data.

The detection rates for ctDNA in GBM patients’ blood also vary (10–55%), highlighting the need for more studies with larger cohorts to better understand ctDNA in GBM. GBM patients often develop resistance to treatment. Monitoring patients over the course of treatment, by serial sampling, and detecting specific tumour mutations and changes in DNA methylation pattern, might prove valuable for understanding tumour behaviour. These parameters might complement the current conventional methodologies used in managing GBM patients. Moreover, multiple collections would enable tumour progression to be monitored, or pseudoprogression to be detected in a minimally invasive manner. When different biofluid sources are compared—for example, ctDNA detected in blood or in CSF—CSF appears to be more representative, possibly owing to the proximity of CSF to the brain. Nevertheless, CSF collection is much more invasive and risky compared with blood collection.

The exosome field in GBM is also emerging and has been producing promising data, such as detection of the EGFRvIII deletion variant in tumour tissue (39.5%) matching with EGFRvIII expression in exosomes (44.7%), and in both cases correlating with poor survival. However, some technical limitations also need to be addressed in the future for this field. In addition, the cohort size of the majority of the studies is small, and currently there are no specific isolation protocols to reliably distinguish EV subtypes.

The idea of detecting CTCs, ctDNA and exosomes that carry predictive markers for GBM, such as IDH1, MGMT and EGFRvIII, is interesting as it can represent a way of getting diagnostic and prognostic information in a non-invasive manner. Because each marker has advantages and disadvantages (see Table 1), a combination of markers might be beneficial. The rapid advances in the field of liquid biopsy have given rise to the investigation of a number of different and complementary biomarkers, which might better inform on the tumour status and present complementary information to treating clinicians when tumour data are lacking or limited, as well as improve molecular stratification of patients for target therapies, and offer information on what therapies might be effective and how to track treatment over time. More studies are needed, with larger cohorts, to increase specificity and sensitivity, and to advance future clinical applications.

**Table 1:** Summary of advantages and disadvantages of using CTCs, ctDNA and exosomes as biomarkers in cancer

	Advantages	Disadvantages	Reference
CTCs	• Information can be provided at the protein, DNA and RNA levels	• CTCs are rare (1 cell in 10^9^ blood cells)	^25,28,47,75^
	• Possibility of carrying out functional assays	• May represent only part of the tumour mass heterogeneity	
	• There are new technologies in the development phase to isolate CTCs	• Process to isolate them is challenging	
ctDNA	• Higher ctDNA levels compared with CTC	• Short half-life, <1.5 h	^23,25,46,75^
	• Levels correlate with disease stage	• Released mainly by cells undergoing necrosis or apoptosis	
	• Easy detection		
Exosomes	• Can be released by all cells, including tumour cells	• The release is not exclusive from tumour cells	^25,115,135^
	• Can carry proteins, DNA, RNA and miRNA	• Possible presence of contaminants by current isolation methods	
	• Present protection for their content		

## Data Availability

Not applicable
